# LYAR promotes colorectal cancer cell mobility by activating galectin-1 expression

**DOI:** 10.18632/oncotarget.5335

**Published:** 2015-09-25

**Authors:** Yupeng Wu, Ming Liu, Zhuchen Li, Xiao-Bin Wu, Ying Wang, Yadong Wang, Min Nie, Feifei Huang, Junyi Ju, Chi Ma, Renxiang Tan, Ke Zen, Chen-Yu Zhang, Keqin Fu, Yu-Gen Chen, Ming-Rong Wang, Quan Zhao

**Affiliations:** ^1^ The State Key Laboratory of Pharmaceutical Biotechnology, School of Life Sciences, Nanjing University, Nanjing, 210046, China; ^2^ Anhui Research Institute for Family Planning, Anhui Research Center for Population and Birth Control, Hefei, 230031, China; ^3^ The Affiliated Hospital of Nanjing University of Chinese Medicine, Nanjing, 210029, China; ^4^ The State Key Laboratory of Molecular Oncology, Cancer Hospital and Institute, Chinese Academy of Medical Sciences, Peking Union Medical College, Beijing, 100021, China

**Keywords:** LYAR, galectin-1, colorectal cancer, cell migration and invasion

## Abstract

Colorectal cancer (CRC) is one of the leading causes of cancer-related death worldwide. However, the molecular mechanisms of CRC pathogenesis are not fully understood. In this study, we report the characterization of LYAR (Ly-1 antibody reactive clone) as a key regulator of the migration and invasion of human CRC cells. Immunohistochemistry analysis demonstrated that LYAR is expressed at a higher level in metastatic CRC tissues. We found that LYAR promoted the migratory and invasive capabilities of CRC cells. Gene expression profile analysis of CRC cells showed that LGALS1, which encodes the galectin-1 protein, was a potential target of LYAR. The ChIP assay and gene reporter assays indicated that LYAR directly bound to the LGALS1 promoter. The ectopic expression of galectin-1 partially restored the mobile potential of LYAR knocked-down cells, which suggests that galectin-1 contributed to the LYAR-promoted cell migration and invasion of CRC cells. Thus, this study revealed a novel mechanism by which the transcription factor LYAR may promote tumor cell migration and invasion by upregulating galectin-1 gene expression in CRC.

## INTRODUCTION

Colorectal cancer (CRC) is the third most common cancer and the fourth most common cause of cancer-related death worldwide, and it accounts for more than half a million deaths per year [1, 2]. Despite advances in diagnostic techniques, neoadjuvant chemoradiotherapy and surgery, the 5-year survival rate for colorectal cancer remains poor, particularly in patients with cancer metastasis [1, 3, 4]. The molecular mechanisms underlying the invasion-metastasis cascade are not fully understood.

LYAR (Ly-1 antibody reactive clone), a 45 kD nucleolar protein, was initially identified from a mouse T-cell leukemia line in 1993 and consists of a zinc finger motif and three nuclear localization signals [5]. Since then, there were few reports about studies on LYAR until recently that LYAR was found to play important roles in human neural tube defects (NTD) [6], spermatogenesis and fertility [7], ribosome biogenesis [8] and translational control [9]. Last year, we demonstrated that LYAR is a transcription factor and has a DNA-binding motif (GGTTAT/G) that represses human fetal globin gene expression in both K562 cells and primary adult human erythroid progenitor cells [10]. A gene expression profile study with whole blood cell mRNA from ovarian cancer patients revealed LYAR and other five genes (PDIA3, NOP14, NCALD, MTSS1 and CYP1B1) as potential prognostic biomarkers for curative and postoperative supportive therapies for ovarian cancer [11]. However, the potential function of LYAR in human cancers, including CRC, is not yet clear.

Galectin-1, which is encoded by LGALS1, is a homodimer of 14 kD subunits, which belongs to a family of soluble galactoside-binding proteins [12]. Galectin-1 is an important multifunctional protein that is involved in cell adhesion, the interaction between cells and the extracellular matrix, cell transformation, proliferation, apoptosis, migration, metastasis, and angiogenesis [12]. Galectin-1 was significantly altered during the development of various malignant tumors and metastases, including colorectal cancer [13–15]. However, little is currently known about the regulatory mechanism of galectin-1 in cancer.

In the present study, we demonstrate that LYAR is a novel key regulator in the progression of colorectal cancer. We show that LYAR activates LGALS1 expression by binding to its promoter to promote CRC cell migration and invasion.

## RESULTS

### LYAR is highly expressed in human CRC tissues and is associated with metastasis in colorectal cancer patients

**Figure 1:**
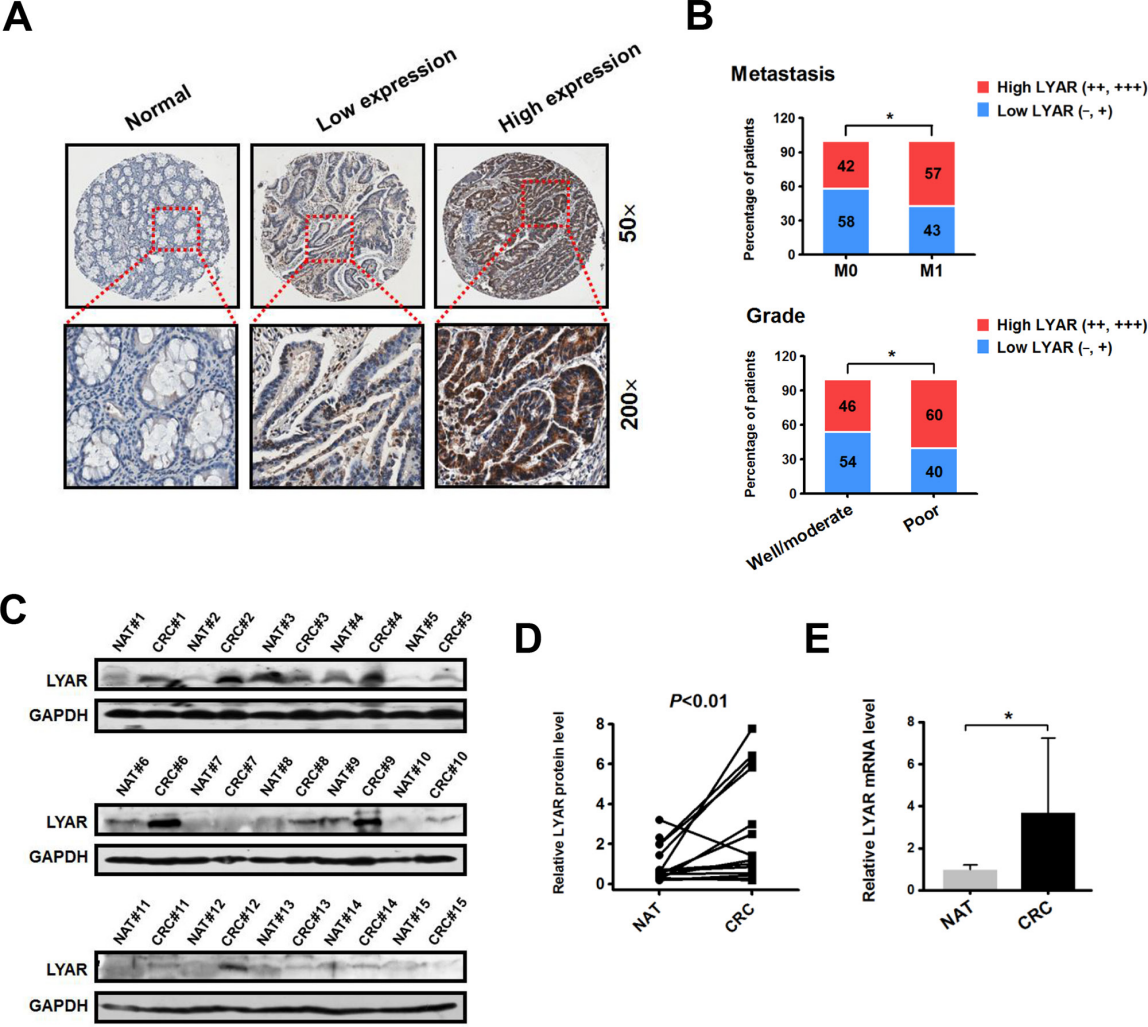
LYAR is highly expressed in human metastatic CRC tissues **A.** Immunohistochemical staining of the LYAR protein in adjacent normal and carcinoma tissues from colorectal cancer patients. Representative micrographs are shown at the original magnification (50 × and 200 × ) as indicated. Low (−, +) and high LYAR expression (++, +++) were classified according to the LYAR immunostaining scores, IRS. **B.** The percentage of patients with different metastasis statuses (M0, no regional or distant metastasis; M1, regional or distant metastasis) and differentiation grades as scored by IRS. *P < 0.05 compared with the indicated group. **C.** Western blot analysis of LYAR in cell lysates from normal adjacent tissues (NAT) and colorectal tumor tissues (CRC) (*n* = 15). GAPDH served as a loading control. **D.** Quantitation of the density of the protein bands from the western blots in (C); *P* < 0.01 compared with the paired NAT. **E.** Quantitation of the LYAR mRNA levels normalized to GAPDH mRNA levels from the tissues from in (C). *P < 0.05 compared with the NAT control.

To examine the LYAR expression levels in CRC, we performed immunohistochemistry (IHC) analysis on tissue arrays of 77 paraffin-embedded adjacent sections of normal colorectal tissue and CRC tissues (Supplementary Table S1). Compared with the adjacent normal colorectal tissues, significantly higher LYAR expression was detected in 49% (38/77) of the total CRC tissues (Figure 1A and Supplementary Table S1). Weak LYAR staining was observed in the early-stage CRC tissues, whereas strong LYAR staining was observed in the advanced-stage CRC tissues (Figure 1A and 1B). Notably, LYAR expression correlated with higher grade tumors and a higher metastasis status (Figure 1B). Western blot analysis of tumor tissues from 15 patients confirmed that the LYAR expression levels in the tumor tissues were significantly higher than those in the matched adjacent normal tissues (Figure 1C and 1D). Quantitative real-time PCR determined that LYAR expression was transcriptionally increased in the CRC tissues compare with the adjacent normal colorectal tissues (Figure 1E). This result is consistent with the data obtained from the Oncomine database, in which LYAR was significantly upregulated in the CRC tissues compared with the adjacent normal tissues (Supplementary Figure S1). Taken together, these results indicate that LYAR was highly expressed in advanced-stage and metastatic CRC patient tissues, which suggests that LYAR expression may be involved in the invasion-metastasis cascade of cancer cells in patients with CRC.

### LYAR promotes CRC cell migration and invasion

**Figure 2:**
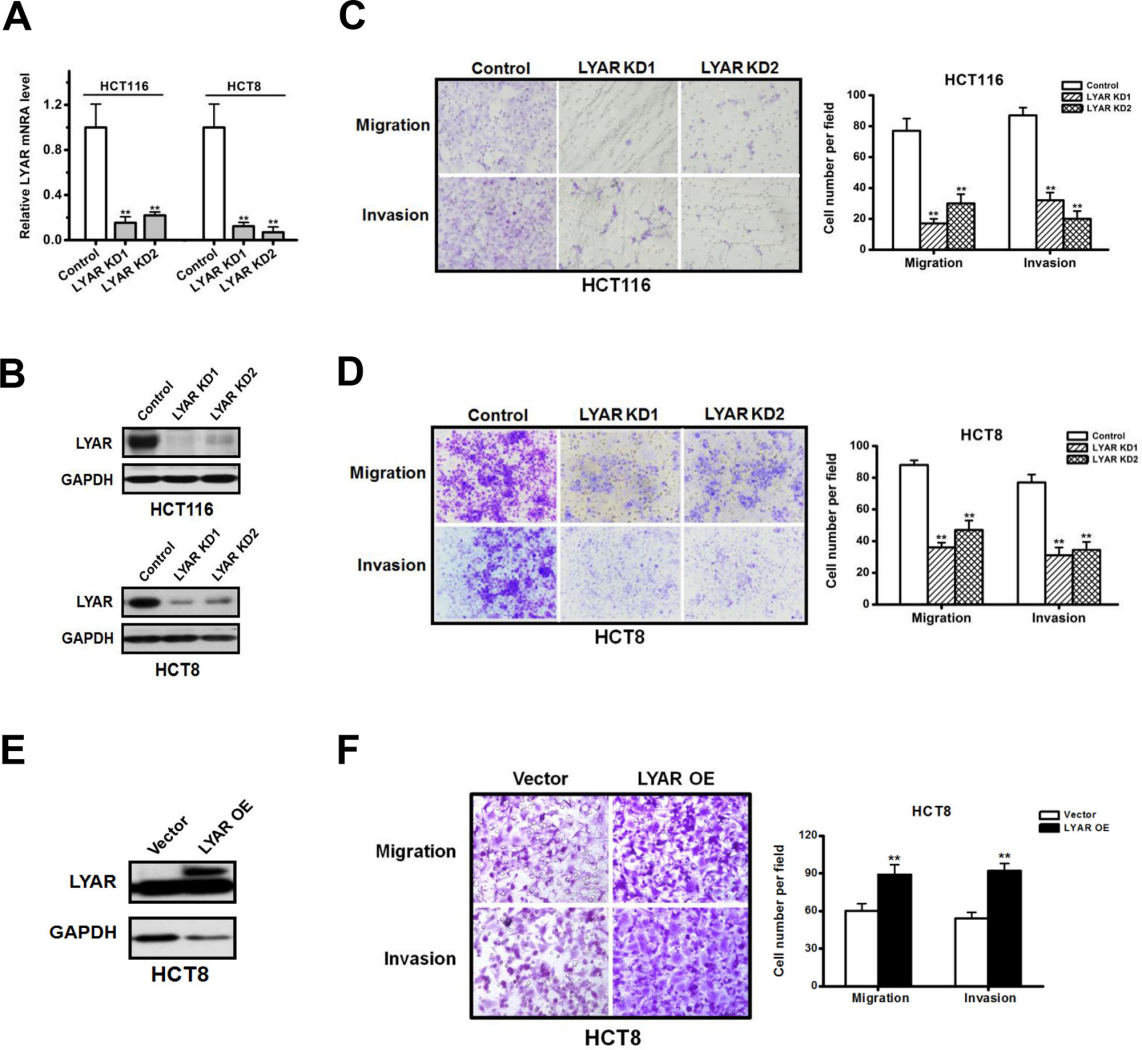
LYAR promotes colorectal cancer cell migration and invasion **A.** Quantitative real-time PCR analysis of LYAR mRNA levels normalized to GAPDH mRNA levels from scrambled control or LYAR-knockdown (KD) HCT116 and HCT8 cells. The results are shown as the mean ± SD from three independent experiments; **P < 0.01 compared with the scrambled control. **B.** Western blot assay showing LYAR protein expression following LYAR knockdown in HCT116 and HCT8 cells. GAPDH served as a loading control. **C.** Effects of LYAR knockdown on cell migration and invasion by Boyden chamber assays in HCT116 cells. Morphologic comparisons of the cells penetrating the artificial basement membrane are shown. The results are shown as the means ± SD from three independent experiments; **P < 0.01 compared with the scrambled control. **D.** Effects of LYAR knockdown on cell migration and invasion by Boyden chamber assays in HCT8 cells. Morphologic comparisons of the cells that penetrated the artificial basement membrane are shown. The results are shown as the means ± SD from three independent experiments; **P < 0.01 compared with the scrambled control. **E.** Western blot assay showing LYAR protein expression following LYAR overexpression in HCT8 cells. GAPDH served as a loading control. **F.** Effects of LYAR-overexpression on cell migration and invasion by Boyden chamber assays in HCT8 cells. Morphologic comparisons of the cells that penetrated the artificial basement membrane are shown. The results are shown as the means ± SD from three independent experiments; **P < 0.01 compared with the scrambled control.

LYAR has been previously shown to associate with cancer potential [5, 11], but the biological function of LYAR in cancer is poorly understood. To explore the function of LYAR in colorectal cancer, we knocked-down LYAR expression in both HCT116 and HCT8 cells with two independent small interfering RNAs. Quantitative RT-PCR revealed that the levels of the LYAR mRNA were reduced to less than 30% of the scrambled control (Figure 2A). Accordingly, Western blot analysis confirmed that the LYAR protein was markedly decreased in the two cell lines (Figure 2B). We then performed cell cycle, apoptosis, cell proliferation and colony formation assays. The results demonstrated that LYAR did not appear to have an impact on these processes (Supplementary Figure S2). In contrast, we observed a significant decrease in the cell migration and invasion potential in the LYAR-knockdown (LYAR-KD) cells compared with the scrambled control cells, which indicated that LYAR could promote cell migration and invasion in colorectal cells (Figure 2C and 2D).

We then analyzed the cell migration and invasion of HCT8 cells in which LYAR was stably overexpressed (LYAR-OE) via the transfection of a lentivirus vector containing the LYAR cDNA (Figure 2E). In contrast to the LYAR-KD cells, the LYAR-OE cells exhibited significant increases in the proportion of migrating and invading cells compared with the vector control cells (Figure 2F). These results suggest that LYAR promoted cell migration and invasion in colorectal cancer cells.

### LYAR activates galectin-1 gene expression

**Figure 3:**
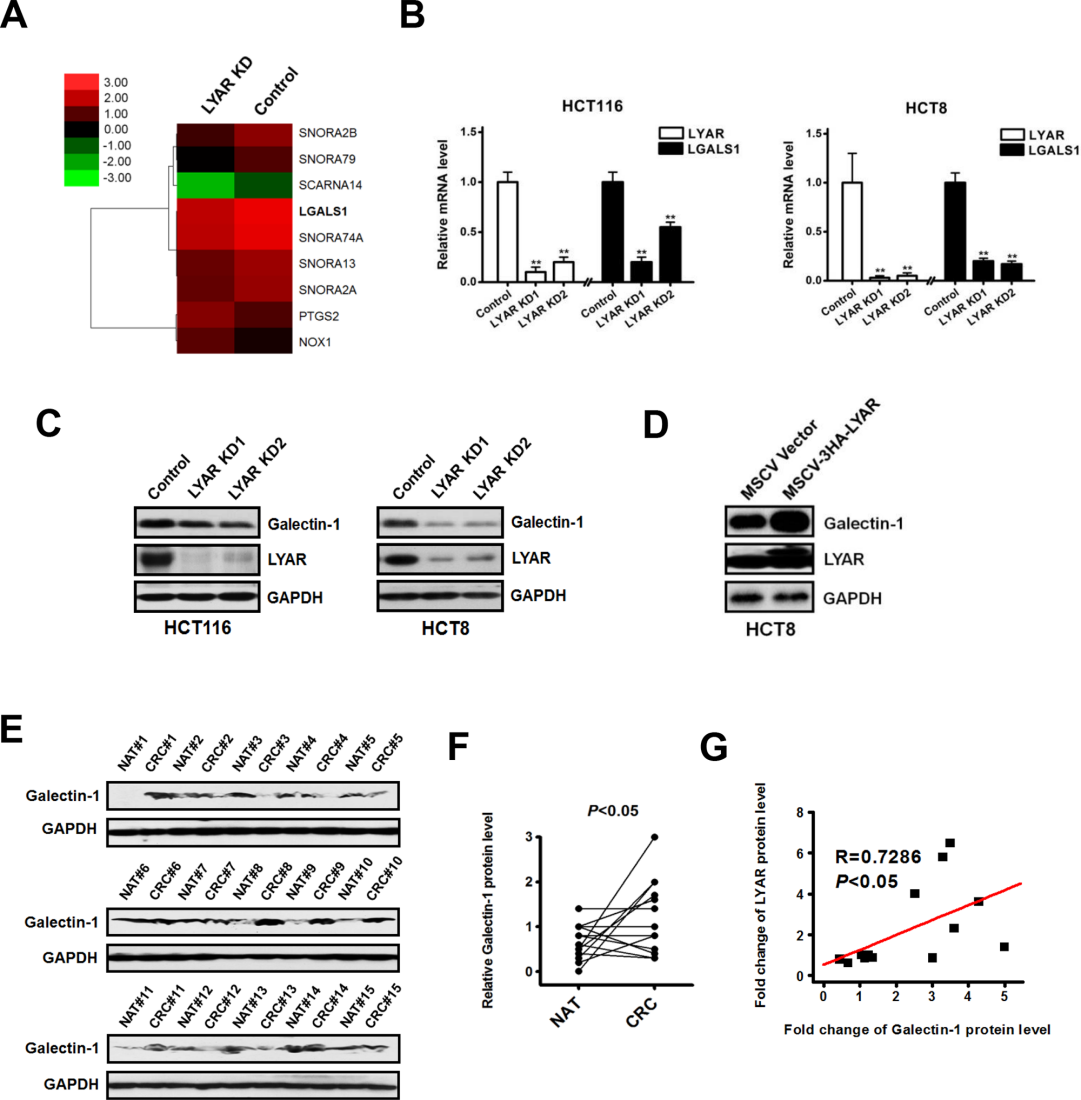
Identification of LGALS1 as a potential LYAR transcriptional target in colorectal cancer cells **A.** Heatmap representation of the microarray data showing the expression levels of nine key migration-related genes in the scrambled control and LYAR-knockdown cells. **B.** Quantitative real-time PCR analysis of LYAR and LGALS1 from LYAR-knockdown or scrambled control HCT116 and HCT8 cells. The results are shown as the means ± SD from three independent experiments; **P < 0.01 compared with the scrambled control. **C.** Western blot assay showing LYAR and galectin-1 protein expression following LYAR knockdown in HCT116 and HCT8 cells. GAPDH served as a loading control. **D.** Western blot assay showing LYAR and galectin-1 protein expression following LYAR overexpression in HCT8 cells. GAPDH served as a loading control. **E.** Western blot analysis of galectin-1 in cell lysates from normal adjacent tissues (NAT) and colorectal tumor tissues (CRC) (*n* = 15). GAPDH served as a loading control. **F.** Quantitation of the density of the protein bands from the western blots in (E); *P* < 0.05 compared with the paired NAT. **G.** Pearson's correlation scatter plot of the fold change in galectin-1 protein levels and LYAR protein levels in human colorectal cancer tissues (*n* = 15, *P* < 0.05).

To address the potential molecular mechanisms by which LYAR may function in colorectal cancer invasion and metastasis, we performed a whole-genome microarray analysis of gene expression in the LYAR knockdown and scrambled control HCT8 cells. We identified 111 upregulated and 129 downregulated genes that had significant changes in their expression levels between the LYAR-KD and scrambled control cells (Supplementary Table S2 and Table S3). Among these differentially expressed genes, nine key genes were associated with invasion and metastasis (Figure 3A) and were thus selected for further evaluation in four CRC cell lines: HCT8, HCT116, LoVo and RKO cells. In these CRC lines, only the expression of LGALS1 was consistently decreased in LYAR-KD cells compared with the scrambled controls according to quantitative RT-PCR (Figure 3B). This result strongly suggests a potential link between LYAR and LGALS1 gene expression in colorectal cancer. In addition, western blot analysis confirmed that LYAR knockdown reduced the expression of galectin-1 protein in four cell lines (Figure 3C and Supplementary Figure S3). Moreover, cells overexpressing LYAR showed a significant increase of galectin-1 expression (Figure 3D). Furthermore, western blot analysis confirmed that the expression levels of galectin-1 in the tumor tissues from 15 patients were significantly higher than in matched adjacent non-tumor tissues (Figure 3E and 3F). Levels of galectin-1 protein and levels of LYAR protein in CRC tissues exhibited a significant correlation calculated by Pearson's correlation (Figure 3G), further indicating that LGALS1 is a potential target of LYAR in cancer cells. Together, these results indicated that LYAR activated LGALS1 gene expression in CRC cells.

### LYAR binds to the LGALS1 promoter in CRC cells

**Figure 4:**
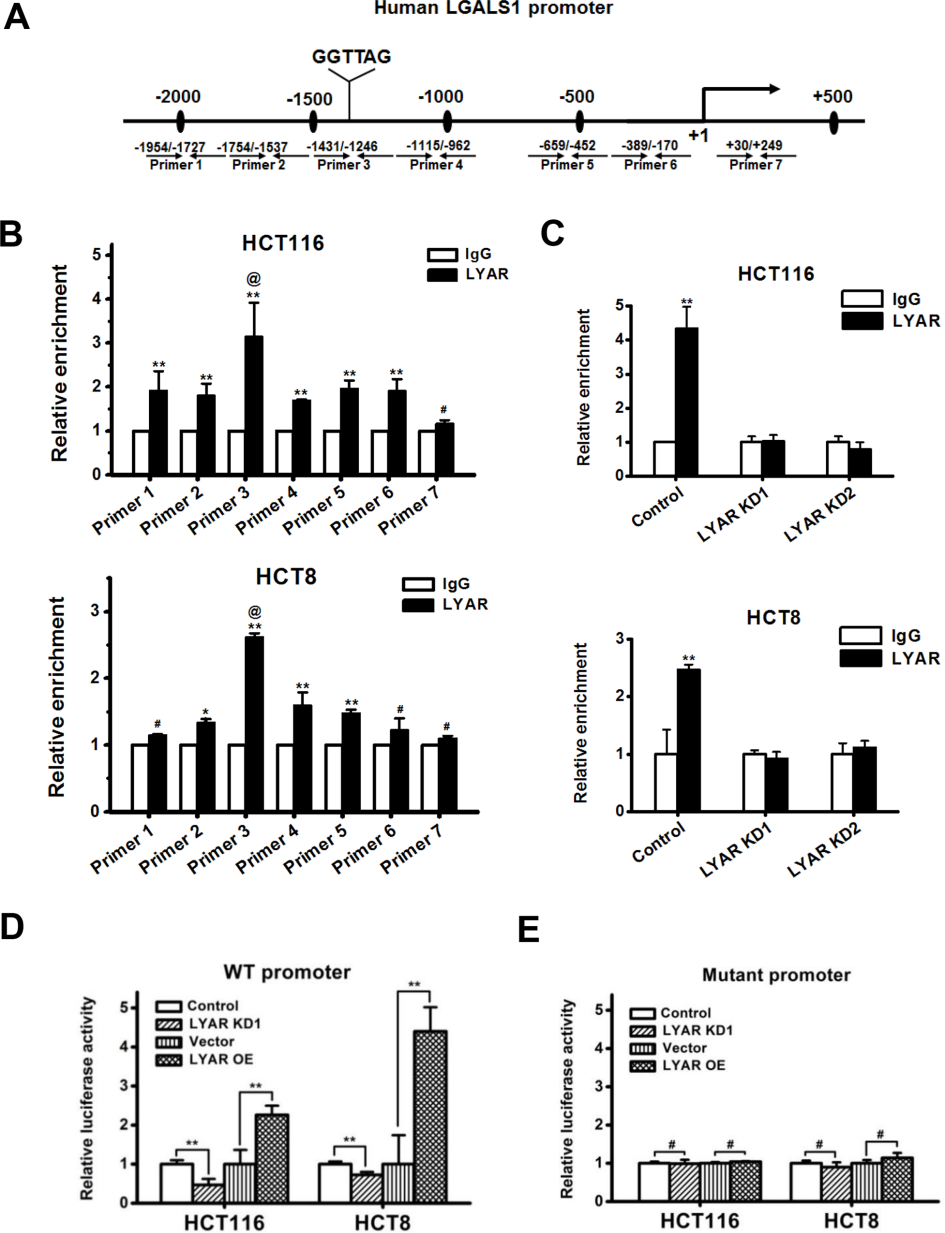
LYAR binds to the LGALS1 promoter to directly activate the expression of LGALS1 **A.** A schematic diagram of the seven primer pairs that span the LGALS1 promoter that were designed for ChIP. **B.** ChIP analysis of LYAR on the LGALS1 promoter in HCT116 and HCT8 cells. Normal rabbit IgG served as a control. The results are shown as the means ± SD from three independent experiments; **P < 0.01, *P < 0.05, ^#^*P* > 0.05 compared with the IgG control. ^@^*P* < 0.05 compared with LYAR enrichment at flanking sites. **C.** ChIP analysis of LYAR on the LGALS1 promoter in LYAR-knockdown HCT116 and HCT8 cells. Normal rabbit IgG served as a control. The results are shown as the means ± SD from three independent experiments; **P < 0.01 compared with the IgG control. **D.** Luciferase reporter analyses of wild-type LGALS1 promoter in LYAR knockdown or overexpressing HCT116 and HCT8 cells. The results are shown as the means ± SD from three independent experiments; **P < 0.01 compared with the scrambled or vector control. **E.** Luciferase reporter analyses of the LGALS1 mutant promoter in LYAR knockdown or overexpressing HCT116 and HCT8 cells. The results are shown as the means ± SD from three independent experiments; ^#^*P* > 0.05 compared with the scrambled or vector control.

We and others have previously demonstrated that LYAR has zinc finger DNA-binding motifs and binds to the DNA at the GGTTAT/G consensus motif [5, 10]. We examined the LGALS1 promoter and found that there was a DNA sequence (CTAACC) at −1359 bp upstream of the LGALS1 gene transcription start site, which is complementary to the consensus DNA-binding motif of LYAR (Figure 4A). To examine whether LYAR could bind to the LGALS1 promoter and directly activate its expression, we performed a ChIP analysis across the promoter and demonstrated that LYAR was indeed the most enriched gene at the promoter region between −1431 and −1246 bp in both HCT116 and HCT8 cells (Figure 4B). Moreover, when LYAR was knocked down in these cells, the LYAR enrichments on the LGALS1 promoter were significantly reduced in both cells (Figure 4C). Furthermore, the gene reporter assay demonstrated that the binding of LYAR to the LGALS1 promoter changed in accord with the LYAR expression levels observed in both HCT116 and HCT8 cells that were transfected with a wild-type promoter-driven luciferase construct (Figure 4D). However, in both HCT116 and HCT8 cells that were transfected with a mutant promoter-driven luciferase construct in which the LYAR consensus motif was mutated from CTAACC to CTGATC, the relative LGALS1 promoter activity did not change, regardless of the changes in the LYAR expression levels (Figure 4E). These results indicated that LYAR directly activated LGALS1 gene expression and that the LYAR-binding motif, CTAACC, on the gene promoter is critical for LYAR-mediated transcription activity.

### Exogenous expression of galectin-1 partially restores mobile potential of LYAR-KD cells

**Figure 5:**
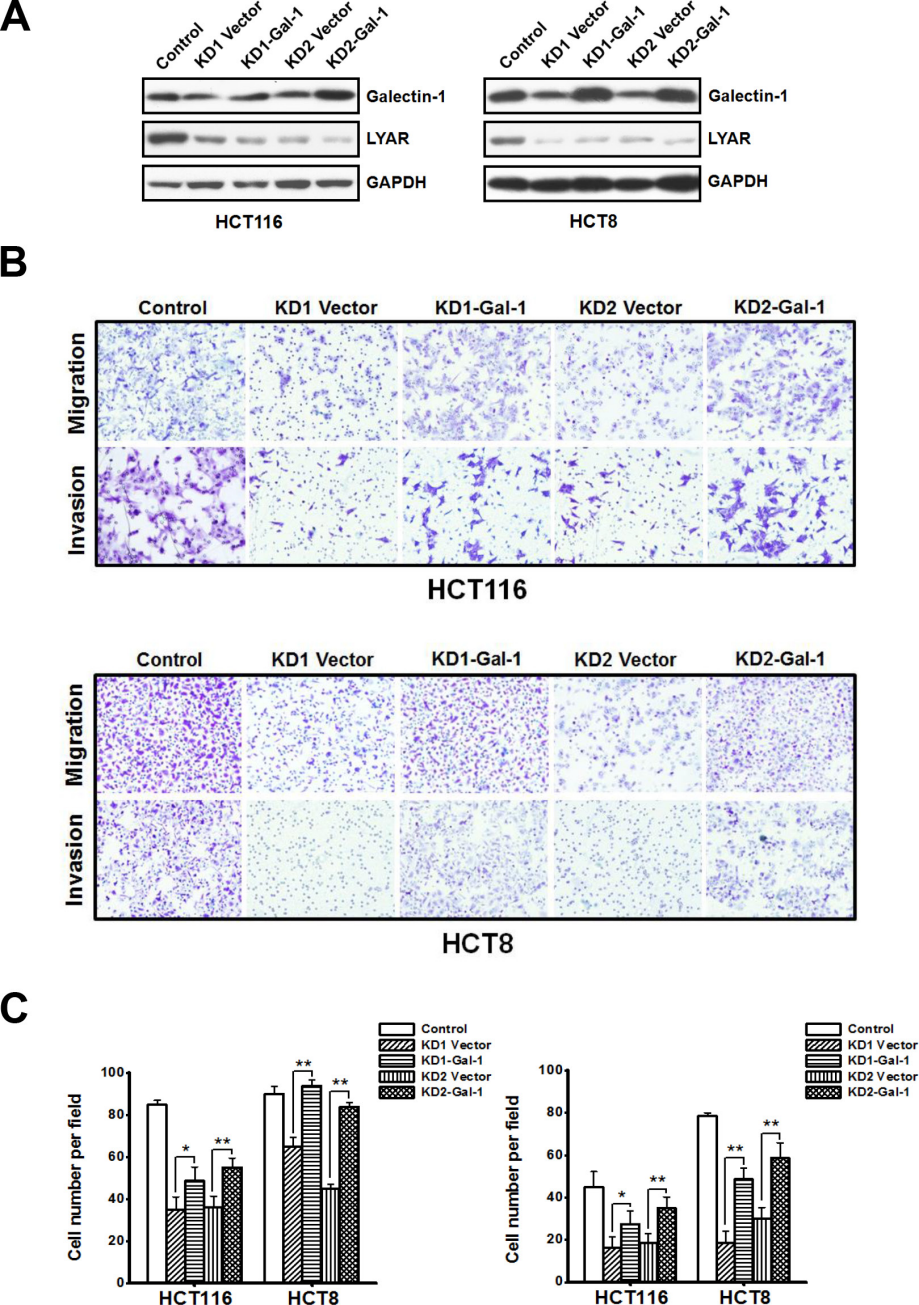
Galectin-1 overexpression restores the mobile potential of LYAR knockdown cells **A.** Western blot assay showing galectin-1 and LYAR protein expression following galectin-1 overexpression in scrambled control and LYAR-knockdown (KD) HCT116 and HCT8 cells. GAPDH served as a loading control. **B.** Effects of galectin-1 expression on cell migration and invasion in LYAR knockdown HCT116 and HCT8 cells by Boyden chamber assays. Morphologic comparisons of the cells penetrating the artificial basement membrane are shown. **C.** Quantitation of the results from (B) are shown as the means ± SD from three independent experiments; **P < 0.01, *P < 0.05 compared with the empty vector control.

Although little is known about the transcriptional regulation of galectin-1 in colorectal cancer (CRC), it has been clearly shown that galectin-1 plays an important role in the regulation of cell migration in colon cancer [16]. We have demonstrated that LYAR could associate with cancer cell invasion and migration. When LYAR was knocked-down in both HCT116 and HCT8 cells, the expression of galectin-1 was down-regulated, and the cell migration and invasion potential were correspondingly decreased. To investigate whether galectin-1 was a key mediator for LYAR-promoted cancer cell invasion and metastasis, we performed rescue experiments in which galectin-1 was overexpressed in LYAR knockdown cells. The exogenous overexpression of galectin-1 and LYAR knockdown were confirmed by western blot analysis in both HCT116 and HCT8 cells (Figure 5A). Compared with the control LYAR-KD cells, the migration and invasion potential of the HCT116 and HCT8 cancer cells overexpressing galectin-1 was significantly increased (Figure 5B and 5C) indicating that exogenous expression of galectin-1 partially restored mobile potential of LYAR-KD cells. These results suggest that galectin-1 plays a critical role in mediating the LYAR-promoted cell migration and invasion in CRC.

## DISCUSSION

In this study, we found that LYAR is highly expressed in CRC tissues and that the LYAR expression levels correlated with advanced-stage and metastatic colorectal cancer tissues. We showed that LYAR promoted CRC cell migration and invasion. LYAR directly activates galectin-1 gene expression and that galectin-1 is a key molecule mediating LYAR-promoted cell mobility. Thus, LYAR appears to be an important molecule that could function in the invasion-metastasis cascade of CRC.

LYAR was originally identified in a mouse T-cell leukemia line [5]. Interestingly, during the proliferation of some normal cells, the LYAR gene was not expressed. Therefore, the expression of LYAR was suggested to be associated with abnormal cell growth [5]. In fact, in radiation-induced thymic leukemias, LYAR was induced in the preleukemic thymocytes and actively maintained in the acute leukemic thymocytes [5]. In NIH-3T3 cells, LYAR overexpression was also found to promote cell proliferation [9]. In neuroblastoma, LYAR was one gene product that correlated with worse clinical outcomes [17]. However, analysis of the whole-genome expression profile of blood cells in ovarian cancer patients showed that LYAR was one of six genes that were significantly down-regulated in the poor prognosis group [11]. This finding indicates that the function of LYAR in cancer could be cancer cell type-dependent. Intriguingly, mutant mice arising from gene-trap insertion in the LYAR gene developed normally, but the growth of MEFs from LYAR heterozygous and homozygous mutant mice was impaired [6]. Remarkably, the majority of LYAR heterozygous and homozygous mutant female mice lacking p53 developed neural tube defects (NTD), which indicated that LYAR could be a candidate gene for in human NTDs and is consistent with its potential function in neuroblastoma.

Thus, to probe the function of LYAR in CRC, we performed various assays, including cell proliferation, cell cycle, apoptosis, and colony formation assays. Unfortunately, LYAR did not have a significant or consistent effect in CRC in any of these analyses, indicating that, in the current setting, LYAR may not affect these cellular processes in CRC. Interestingly, we found that LYAR indeed played an important role in CRC cell migration and invasion.

Using gene array analysis, we have identified LGALS1 as a potential direct target of LYAR in CRC cells. Galectin-1, encoded by LGALS1, has been identified in many species, with 15 family members so far [12]. Although galectin-1 is normally found at the cell surface, it is also localized to the cell nucleus and cytoplasm and can be secreted to the extracellular matrix [12]. Galectin-1 has been associated with a variety of physiological cell functions; it was shown to be important for tumor development and metastasis and has been associated with cell adhesion, invasion, angiogenesis and the immune response [12, 18]. In fact, galectin-1-knock out mice showed insufficient tumor angiogenesis during tumor growth *in vivo* [19]. Increased levels of galectin-1 have been linked to tumor cell migration and invasion [12, 20, 21]. However, to our knowledge, few studies have showed how galectin-1 is transcriptionally regulated. In human CRC cells, two hypoxia inducible factor-1α (HIF-1α) binding sites were identified −441 to −423 bp upstream to the transcriptional start site of LGALS1 gene and were associated with the hypoxia-mediated migration/invasion of CRC cells [22]. Interestingly, CCAAT/enhancer binding protein α(C/EBPα) physically interacts with HIF-1α and directly binds to the −48 to −42 bp region to activate the expression of galectin-1 and control the differentiation of human acute myeloid leukemia cells [23]. In peripheral blood mononuclear cells, NF-κB was demonstrated to functionally bind to the first intron of the LGALS1 gene to stimulate its gene expression, which can attenuate NF-κB activation and negatively control NF-κB signaling [24]. In mouse embryonal carcinoma cells, the Sp1 binding site at −62 to −41 bp of the LGALS1 gene was shown to be critical for its induction by butyrate [25]. In our current study, we demonstrated that LYAR directly binds to the LGALS1 gene promoter at −1359 bp to induce galectin-1 expression and to promote tumor invasion and the migration of CRC cells. Analyzing the promoter mutants revealed that the DNA sequence CTAACC (complementary to LYAR binding motif, GGTTAG) at −1359 bp on the gene promoter was critical for LGALS1 activation by LYAR. LYAR has become a new member that participates in the transcriptional regulation of LGALS1 gene expression. It is not clear that how LYAR increases the transcription of the LGALS1 gene. It's possible that LYAR works through recruitment of other transcription factors or epigenetic modifiers such as histone acetyltransferases or methyltransferases.

Taken together, this study demonstrated that LYAR promoted the migratory and invasive capabilities of CRC cells. We showed that LGALS1, which encodes galectin-1 protein, was a potential direct target of LYAR. Thus, a novel role for the transcription factor LYAR in CRC has been revealed: LYAR promotes tumor migration and invasion by upregulating galectin-1 gene expression. These findings may help provide a new therapeutic candidate for the treatment of CRC and thus increase the potential survival of CRC patients.

## MATERIIALS AND METHODS

### Tissue microarrays and immunohistochemistry

CRC tissue arrays were purchased from Shanghai Biochip Co., Ltd., Shanghai, China. The clinicopathological characteristics of the samples were listed in Supplementary Table S1. The tissue array slides were deparaffinized in xylene, rehydrated in 100%, 95%, and 75% ethanol and then the slides were immersed and heated in 10 mmol/L citrate buffer (pH 6.0) for antigen retrieval. Endogenous peroxidase was quenched with a 3% hydrogen peroxide solution. Subsequently, non-specific binding was blocked by pre-incubation with 10% goat serum in 1 × PBS. The slides were probed with LYAR primary antibodies [10] at 4°C overnight, and incubated with an anti-rabbit secondary antibody followed by a DAB Kit (Life Technologies). The tissue array slides were counterstained with hematoxylin and photographed on digital pathology system from Aperio ImageScope (Aperio Technologies, Inc.).

Immunohistochemical (IHC) stains were scored semi-quantitatively according to the intensity and percentage of immunoreactive cells (immunoreactive score, IRS system). Immunohistochemical staining of the tissues was scored using the semi-quantitative immunoreactivity score (IRS) independently by two pathologists blinded to the clinical data. Category A documented the intensity of immunostaining as 0–3 (0, negative; 1, weak; 2, moderate; 3, strong). Category B documented the percentage of immunoreactive cells as 1 (0–25%), 2 (26–50%), 3 (51–75%), or 4 (76–100%). Multiplication of the category A score and category B score for each tumor or non-tumor generates an IRS ranging from 0 to 12. Based on the IRS score, the immunoreactivity was classified as follows: negative − (IRS 0–2); positive + (IRS 3–4), ++ (IRS 5–8) and +++ (9–12). Samples with IRS ≤ 4 or IRS > 4 were classified as low or high expression, respectively, of LYAR in tumors.

### Cell culture and western blot assay

The HCT8, LoVo and RKO human colon cancer cell lines were a kind gift from the State Key Laboratory of Molecular Oncology, Cancer Hospital and Institute, Chinese Academy of Medical Sciences, Peking Union Medical College, Beijing, China. The HCT116 human colon cancer cell line was purchased from the Typical Culture Preservation Commission Cell Bank, Chinese Academy of Sciences. HCT8, LoVo and RKO cells were maintained on gelatinized 10-cm plates in RPMI-1640 Medium (Gibco) supplemented with 10% fetal bovine serum (Gibco), 100 U penicillin and 100 mg streptomycin (Gibco) at 37°C and 5% CO_2_. HCT116 cells were cultured in McCoy's 5a Medium (Gibco).

Western blot analysis of the cellular extracts was performed as described previously [10]. The specific primary antibodies used were LYAR [10], galectin-1 (Bio Basic Inc.), HSP70 (Santa Cruz Biotechnology) or GAPDH (MBL International Inc.).

### LYAR silencing by transient transfection of siRNAs

HCT8 cells were transfected with 100–200 pmol of the appropriate siRNA (LYAR-KD and non-silencing control) using 5–10 μL Lipofectamine™ 2000 (Life Technologies) in a 6-well plate. After 48 hours, the cells were collected for cell proliferation, cell cycle, apoptosis, colony formation, adhesion, migration and invasion assays. The siRNA sequences were Human LYAR-siRNA1: 5′-GGGAGGUGAAGAAGAAUAA-3′ Human LYAR-siRNA2: 5′-GCACUCGGAAGUUGAAACA-3′

### Microarray analysis

Approximately 1 × 10^7^cells were collected and treated with Trizol reagent (Life Technologies) in an RNase-free tube. The RNA isolation from the cells, microarray analysis, data processing, statistical analysis and gene ontology analysis were performed by the Shanghai Biotechnology Corporation, Shanghai, China. The microarray experiments were performed using the Affymetrix GeneChip Human Transcriptome Array 2.0 (HTA2.0).

### Plasmid construction and viral infection

To build stable LYAR-KD cells, the LYAR siRNA target sequences and non-silencing sequence were inserted into the *Xho* I/*Hpa* I sites in the pLentiLox 3.7 vectors. To overexpress LYAR, the human LYAR coding sequence (CDS) was cloned into the MSCV-HA-IRES-GFP plasmid at the *Xho* I and *BamH* I sites. To overexpress galectin-1 in LYAR-KD cells, the human LGALS1 cDNA was cloned into the pLVX-IRES-mCherry vector at the *Xba* I and *BamH* I sites. Lentiviruses or retroviruses were packaged and produced in 293T cells. The viral supernatant was collected and filter-sterilized to infect the corresponding cells. The stably transfected cells were sorted and collected by flow cytometry using GFP or mCherry fluorescence.

### Cell migration and invasion assays

Equal amounts of cells (5 × 10^4^ in 200 μL) were seeded into the upper chamber of the transwell apparatus (Corning Costar) in serum-free medium, and medium supplemented with 15% FBS was added to the bottom chamber. After 24–48 h, the cells on the upper surface that did not pass through the 8-μm pore-size polycarbonate filter were removed using a moistened cotton swab; the cells migrating to the lower membrane surface were fixed with 100% methanol, stained with 0.4% crystal violet and counted under a microscope (Nikon). The invasion assay was performed as described in the migration assay, except that the upper chamber was precoated with 50 μL of a Matrigel solution.

### Quantitative RT-PCR

Total RNA was isolated from cells with Trizol reagent (Life Technologies). The cDNAs were generated using the PrimeScript™ RT Master Mix (Perfect Real Time) kit (TaKaRa). The quantitative reverse-transcriptase PCR (Q-RT PCR) primers were selected from the PrimerBank (see http://pga.mgh.harvard.edu/primerbank/) or designed online on the Primer3 website (http://bioinfo.ut.ee/primer3-0.4.0/primer3). Q-RT PCR was performed in a Rotorgene 6000 using the FastStart Universal SYBR Green Master (Rox) (Roche) in a final volume of 20 μL. The relative quantification was performed for genes listed below, and ACTB served as an internal reference in colorectal cancer cells. Each reaction was performed in duplicate and repeated at least three times.

The primers for human ACTB were forward 5′-AGCACAGAGCCTCGCCTT-3′ and reverse 5′-CTC GTCGCCCACATAGGAAT-3′.

The primers for human LYAR were forward 5′-GGAGGCACTCGGAAGTTGAAA-3′ and reverse 5′-GTTCCTCTTCGGATCTGTGATG-3′.

The primers for human LGALS1 were forward 5′-CTGTGCCTGCACTTCAACC-3′ and reverse 5′-CAT CTGGCAGCTTGACGGT-3′.

### Gene reporter and luciferase assay

The LGALS1 promoter region, a 1711-bp sequence that included the specific DNA-binding motif for LYAR, was amplified by PCR from the genomic DNA. Subsequently, the obtained DNA fragment was subcloned into pGL3-Basic (Promega) to construct a luciferase reporter plasmid, and the sequence was confirmed. The luciferase reporter plasmid in which the LYAR binding site was mutated was constructed using the Muta-direct™ site-directed mutagenesis kit (SBS Genetech). Luciferase reporter assays were performed as described previously [28].

### Chromatin immunoprecipitation (ChIP)

ChIP assays were performed as described as previously [29]. Chromatin fractions from HCT8 and HCT116 cells were immunoprecipitated with specific antibodies against LYAR. Normal rabbit immunoglobulin G (IgG, Beyotime) served as the control. The ChIP samples were analyzed by quantitative real-time PCR using the FastStart Universal SYBR Green Master Mix (Roche) and specific primers (Supplementary Table S4) across the LGALS1 promoter. A standard curve was prepared for each set of primers using serial titrations of the input DNA. The percentage of ChIP DNA was calculated relative to the input DNA from primer-specific standard curves using the Rotor-Gene 6000 Series Software 1.7. Each experiment was performed at least two independent times. The following specific primers used were used:

forward, 5′-CCATGCCCAGCTAATTTTGT-3′ and

reverse, 5′-GGTGGAATCTGACCACAACC-3′

### Statistical analysis

Student's *t*-test was used to derive the significance of the differences between the mean values.

## SUPPLEMENTARY FIGURES AND TABLES


