# Supplementary material for: LYAR promotes colorectal cancer cell mobility by activating galectin-1 expression
Source: Oncotarget. 2015 Sep 25;6(32):32890–901. doi: 10.18632/oncotarget.5335 (PMC4741737; doi:10.18632/oncotarget.5335)
Supplement: Supplementary file 1 [file oncotarget-06-32890-s001.pdf]

SUPPLEMENTARY FIGURES AND TABLES

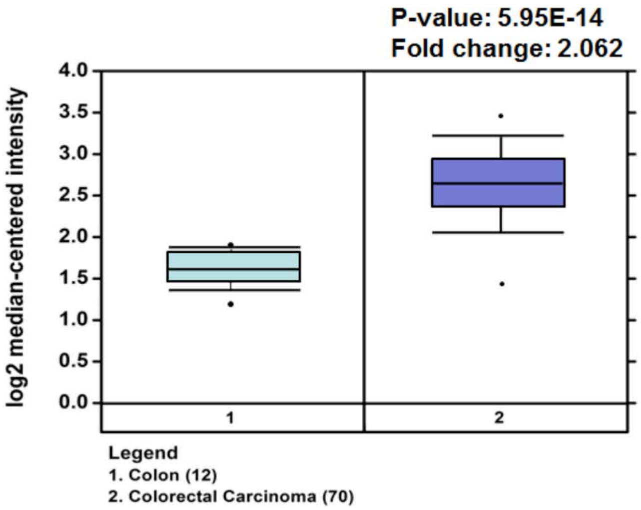

Supplementary Figure S1: LYAR mRNA levels in colorectal carcinoma and normal colon tissues from the Oncomine database (http://www.oncomine.org).

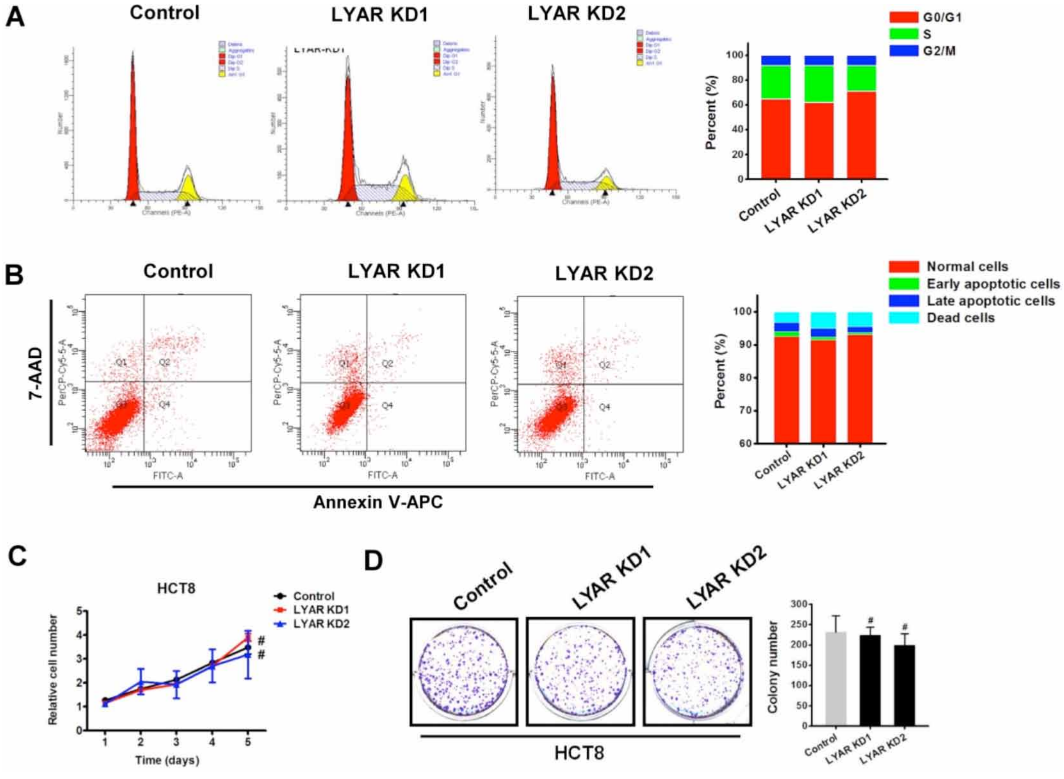

Supplementary Figure S2: LYAR knockdown has no effect on the cell cycle A., apoptosis B., cell proliferation C., and colony formation D. of colorectal HCT8 cells. $^{\#}P > 0.05$ compare to the scrambled control.

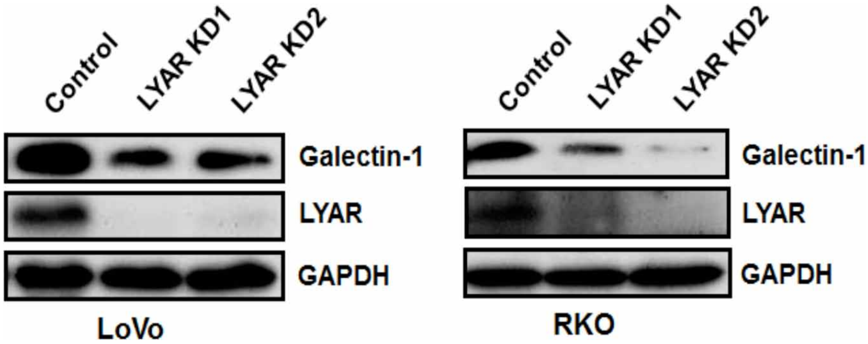

Supplementary Figure S3: LYAR knockdown decreases Galectin-1 expression in LoVo and RKO cells. Western blot assay showing LYAR and Galectin-1 protein expression following LYAR knockdown in Lovo and RKO cells. GAPDH serves as a loading control.

Supplementary Table S1: Characteristics of LYAR expression in CRC patients

		LYAR	
**Clinicopathological feature**	**Total**	**Low**	**High**
	77	39 (51%)	38 (49%)
Age			
< 60	47	27	20
≧60	30	12	18
Gender			
Male	49	23	26
Female	28	16	12
Grade			
Good/moderate	57	31 (54%)	26 (46%)
Poor	20	8 (40%)	12 (60%)
pM			
M0	40	23 (58%)	17 (42%)
M1	37	16 (43%)	21 (57%)
Colon cancer	21	5	16
Rectal cancer	56	34	22

pM, distant metastasis; M0, no regional or distant metastasis; M1, regional or distant metastasis.

Supplementary Table S2: List of the top 111 up-regulated genes following LYAR knockdown

Gene symbol	mRNA accession	Fold change
NOX1	NM_007052	2.10
PTGS2	NM_000963	2.02
CLDN2	NM_001171092	1.92
MSANTD4	NM_032424	1.91
MGAT5	NM_002410	1.89
ANKRD52	NM_173595	1.89
TMEM2	NM_013390	1.87
IFNGR1	NM_000416	1.86
SKP2	NM_001243120	1.85
ARRDC3	NM_020801	1.85
PAPSS2	NM_001015880	1.83
REG4	NM_001159352	1.81
USP38	NM_032557	1.80
LOC100507039	ENST00000433933	1.79
LOC100507376	ENST00000513179	1.79
HSD3B1	NM_000862	1.76
DCBLD2	NM_080927	1.75
C1QTNF9B-AS1	NM_001135816	1.74
PGBD4	NM_152595	1.73
LGR5	NM_003667	1.73
WDR5B	NM_019069	1.73
LOC100134229	NR_024451	1.73
GBP3	NM_018284	1.73
OR10H2	NM_013939	1.72
RSL24D1P11	ENST00000440667	1.70
ME1	NM_002395	1.70
PLK2	NM_001252226	1.69
EID2B	NM_152361	1.69
MIR32	NR_029506	1.68
PTPRJ	NM_001098503	1.67
ANXA1	NM_000700	1.67
HEPH	NM_001130860	1.67
HMGCS2	NM_001166107	1.67
C2orf44	NM_001142319	1.66
LOC648809	NR_036652	1.65
EFTUD1	NM_001040610	1.65

Gene symbol	mRNA accession	Fold change
PTPLAD1	NM_016395	1.65
MUT	NM_000255	1.65
ZW10	NM_004724	1.65
SGPP1	NM_030791	1.64
E2F7	NM_203394	1.64
MOSPD1	NM_019556	1.64
HSDL2	NM_001195822	1.64
IFIT5	NM_012420	1.63
CYP39A1	NM_016593	1.63
SERINC1	NM_020755	1.63
C6orf47	NM_021184	1.62
ERVH48–1	ENST00000447535	1.62
LOC389641	NR_033928	1.61
WNK1	BC130469	1.61
KDELC2	NM_153705	1.61
MORC4	NM_024657	1.61
CD164	NM_001142401	1.61
TUG1	NR_002323	1.60
GTF2H1	NM_001142307	1.60
BMPR1APS2	ENST00000451822	1.60
RNU7–72P	ENST00000516314	1.60
C1QTNF9-AS1	ENST00000449656	1.59
C6orf120	NM_001029863	1.59
IGFL4	NM_001002923	1.59
GPR42	ENST00000454971	1.59
MTF2	NM_001164391	1.59
SNRPEP4	ENST00000435815	1.59
DDX6	NM_004397	1.58
ABCB1	NM_000927	1.57
CDH17	NM_001144663	1.57
PITPNA-AS1	NR_028514	1.57
TSPAN8	NM_004616	1.57
IGFL2	NM_001002915	1.57
MDM2	NM_002392	1.57
MED11	NM_001001683	1.56
TP53INP1	NM_001135733	1.56
LOC728819	NM_001101330	1.55
APPBP2	NM_006380	1.55

Gene symbol	mRNA accession	Fold change
LACTB	NM_032857	1.55
RAB27B	NM_004163	1.55
MAP3K2	NM_006609	1.55
TRIM31-AS1	ENST00000440874	1.55
SAT1	NM_002970	1.55
ATP8B1	NM_005603	1.55
IL33	NM_001199640	1.55
BMPR1A	NM_004329	1.55
GCSHP3	NR_033248	1.54
LGALS8	NM_006499	1.54
ARRDC4	NM_183376	1.54
USP27X	NM_001145073	1.54
YEATS4	NM_006530	1.53
DCP2	NM_001242377	1.53
NT5E	NM_001204813	1.53
SLC12A2	NM_001046	1.53
PIGN	NM_012327	1.53
LOC100506676	ENST00000413077	1.53
RSPRY1	NM_133368	1.53
CADPS	NM_003716	1.53
ANKRD49	NM_017704	1.52
MIR3936	NR_037500	1.52
LINC00460	NR_034119	1.52
PLDN	NM_012388	1.52
ZNF543	NM_213598	1.52
CNOT6LP1	ENST00000393358	1.52
GALNT8	NM_017417	1.52
STX12	NM_177424	1.51
GDF11	NM_005811	1.51
LOC100129361	NR_036583	1.51
SCML1	NM_001037535	1.51
G3BP2	NM_012297	1.50
MRFAP1L1	NM_203462	1.50
BCL2L15	NM_001010922	1.50
LINC00339	NR_023918	1.50
FAM103A1	NM_031452	1.50
LRRC40	NM_017768	1.50

Supplementary Table S3: List of the top 131 down-regulated genes following LYAR knockdown

Gene symbol	mRNA accession	Fold change
MCCD1	NM_001011700	0.65
RN5S263	ENST00000363376	0.65
CES2	NM_003869	0.65
LOC100505550	ENST00000445172	0.65
LOC646903	NR_036538	0.65
RN5S435	ENST00000364044	0.65
RN5S141	ENST00000364543	0.65
MIR422A	ENST00000362286	0.65
SNORD12	NR_003030	0.65
RN5S188	ENST00000362916	0.65
RN5S336	ENST00000364189	0.65
RN5S506	ENST00000364816	0.65
SNORD14E	ENST00000364009	0.65
RN5S270	ENST00000411361	0.65
RN5S265	ENST00000411023	0.65
SNORA30	NR_002966	0.64
SNORD1B	NR_004396	0.64
RNY3P3	ENST00000363816	0.64
MIR4462	NR_039669	0.64
SNORA23	NR_002962	0.64
RN5S134	ENST00000516492	0.64
TERC	NR_001566	0.64
RNU4ATAC	NR_023343	0.64
LOC100130776	NR_027032	0.64
MIRLET7F2	NR_029484	0.64
LOC100287404	ENST00000515566	0.64
RN5S308	ENST00000362532	0.64
RN5S449	ENST00000516871	0.64
USP17	NM_001105662	0.64
USP17	NM_001632	0.64
ALPP	ENST00000364914	0.64
SNORD14A	NR_045370	0.64
LOC100505815	NM_032108	0.64
SEMA6B	ENST00000453284	0.63
ZNF32-AS1	NR_026818	0.63
FAM138A	NM_021049	0.63

Gene symbol	mRNA accession	Fold change
MAGEA5	ENST00000365378	0.63
RN5S85	ENST00000364931	0.63
RNU5E-4P	NR_002977	0.63
SNORA45	NM_017797	0.63
BTBD2	NR_039684	0.63
MIR4473	ENST00000364491	0.63
RN5S433	NM_001164453	0.63
FAM90A20	NR_000027	0.63
SNORD83A	ENST00000517249	0.63
RN5S437	NM_001007189	0.63
IGIP	ENST00000411404	0.63
RNU2–6P	NR_003022	0.63
SNORA59B	NR_003022	0.63
SNORA59B	ENST00000362698	0.63
RNU5A-1	NM_001362	0.62
DIO3	NR_002960	0.62
SNORA20	ENST00000364171	0.62
RN5S242	ENST00000516501	0.62
RNY5	NR_002913	0.62
SNORD63	ENST00000516076	0.62
RN5S381	NR_000024	0.62
SNORD46	ENST00000383970	0.61
RNY1P8	NM_181623	0.61
KRTAP15–1	NR_030173	0.61
MIR432	ENST00000364477	0.61
RN5S69	NR_029422	0.61
RNU12	NM_006834	0.61
RAB32	ENST00000362918	0.61
RNY3P2	NR_000025	0.61
SNORD15B	ENST00000365050	0.61
RN5S442	ENST00000410495	0.61
RN5S338	NR_003005	0.61
SCARNA4	NM_001242791	0.61
FLJ45513	NM_001013653	0.61
LRRC26	NM_030876	0.61
OR5V1	NR_036146	0.60

Gene symbol	mRNA accession	Fold change
MIR548W	NR_003029	0.60
SNORD9	NR_003066	0.60
SNORD85	NR_024277	0.60
AGPAT4-IT1	ENST00000390590	0.60
IGHD3–3	ENST00000516081	0.60
RN5S497	NM_001014291	0.60
SPRR2G	NM_001029891	0.60
PGAM4	ENST00000516887	0.60
RN5S34	NR_003015	0.60
SNORA53	NM_000755	0.59
CRAT	ENST00000516442	0.59
RN5S348	NR_002997	0.59
SCARNA1	NR_003014	0.59
SNORA47	ENST00000363603	0.59
RN5S507	ENST00000516707	0.59
RN5S82	NR_003031	0.59
SNORD11	NM_019106	0.58
ATP1A3	NR_002974	0.58
SNORA42	ENST00000429561	0.58
SHANK2-AS1	NR_002953	0.58
SNORA11	ENST00000385293	0.57
MIR616	NM_001142776	0.57
CHAC1	ENST00000414156	0.57
ERI3-IT1	NR_002919	0.57
SNORA5A	NR_038970	0.56
LOC283624	ENST00000363245	0.56
RN5S440	NR_003007	0.56
SCARNA23	ENST00000516712	0.56
RNU5D-2P	NR_002921	0.56
SNORA75	NM_007028	0.56
TRIM31	ENST00000363286	0.55
RNU5B-1	NM_005860	0.55
FSTL3	NR_002964	0.55
SNORA28	NR_002576	0.55
SNORA21	NR_031751	0.55
MIR2117	NR_002449	0.54
SNORA65	NR_002585	0.54
SNORA52	NR_004398	0.54

Gene symbol	mRNA accession	Fold change
SNORD82	NM_005323	0.54
HIST1H1T	NR_003058	0.54
SNORD70	NM_006688	0.53
C1QL1	NM_031313	0.53
ALPPL2	ENST00000364533	0.53
SNORD14B	NM_004157	0.52
PRKAR2A	NM_005893	0.52
CCIN	ENST00000411791	0.52
FAM25E	ENST00000426412	0.51
FAM25D	NM_001145725	0.51
**LYAR**	NR_000005	0.51
SNORD15A	NR_002950	0.51
SNORA2A	NR_002922	0.50
SNORA13	NR_004388	0.50
SCARNA14	NR_002754	0.49
RNU5E-1	NR_002915	0.48
SNORA74A	NM_002305	0.48
**LGALS1**	NR_003021	0.46
SNORA79	NR_002951	0.44

Supplementary Table S4: ChIP primer sequences for the LGALS1 promoter

Locus	Forward	Reverse
Primer 1:	TGCCACAGTGATCTTCCCTA	GGCAGAAGCTCCAGAATGAG
Primer 2:	CTGGCCTTCTCATTCTGGAG	AGCCTGGGCAATAAGAGTGA
Primer 3:	CCATGCCCAGCTAATTTTGT	GGTGGAATCTGACCACAACC
Primer 4:	GGGCCTCAGAGGAGATGTTA	TAGAGACGGGGTTTCACCAC
Primer 5:	CAGGGCGCTTTGACTATCC	GAGGGCCACTGACTTAACCA
Primer 6:	CCAGCTCTGATTCCCTGAAG	CAGAGTCCAAGCTCCACCTC
Primer 7:	CATCTCTCTCGGGTGGAGTC	GCCACTGAGGTCCAGAAAAG
